# Supplementary material for: Proline–Proline Dyad in the Fusion Peptide of the Murine β–Coronavirus Spike Protein’s S2 Domain Modulates Its Neuroglial Tropism
Source: Viruses. 2023 Jan 12;15(1):215. doi: 10.3390/v15010215 (PMC9865228; doi:10.3390/v15010215)
Supplement: Supplementary file 1 [file viruses-15-00215-s001.zip › viruses-2007031-supplementary.pdf]

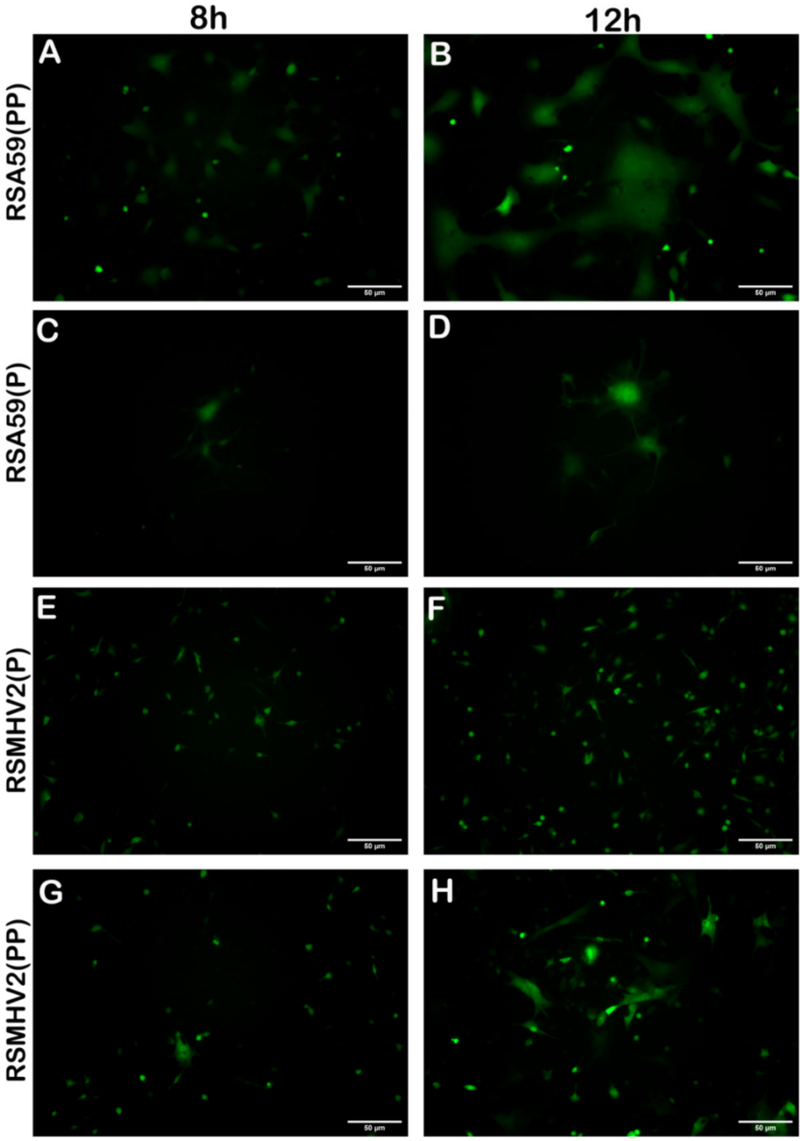

Figure S1. Viral fusion kinetics of RSA59(PP), RSA59(P), RSMHV2(P), and RSMHV2(PP) in primary microglia culture. Primary microglia cultures were infected with RSA59(PP), RSA59(P), RSMHV2(P), and RSMHV2(PP) at MOI 2. The EGFP fluorescent which indicates the viral antigen, from culture plate was monitored under epifluorescent microscopy at interval of 4h. Syncytia formation was observed in 8h p.i. (**A**) and 12h p.i. (**B**) of RSA59(PP) infected cultures. Few syncytia were seen in 8h p.i. (**C**) and 12h p.i. (**D**) of RSA59(P) infected cultures. RSMHV2(P) infected cultures did not show syncytia at both 8h and 12h p.i. (**E–F**) whereas RSMHV2(PP) infection showed syncytia at 8h p.i. (**G**) and 12h p.i. (**H**). Experiments were performed three times.

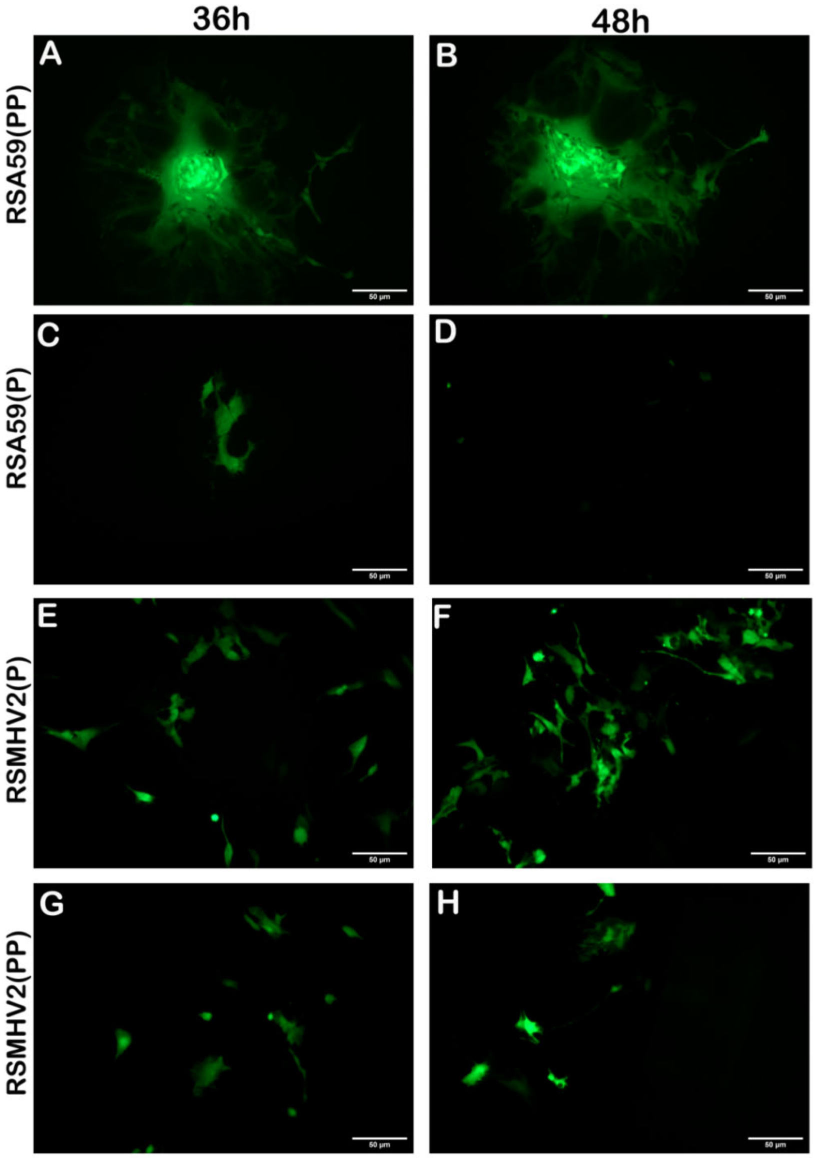

Figure S2. Viral fusogenic kinetics of RSA59(PP), RSA59(P), RSMHV2(P), and RSMHV2(PP) in primary astrocyte culture. Primary astrocyte cultures were infected with RSA59(PP) (**A–B**), RSA59(P) (**C–D**), RSMHV2(P) (**E–F**), and RSMHV2(PP) (**G–H**) at MOI 2. The EGFP fluorescent which indicates the viral antigen, from culture plate was monitored under epifluorescent microscopy at interval of 12h. Giant syncytia was observed in 36h p.i. (**A**) and 48h p.i. (**B**) of RSA59(PP) infected cultures. No syncytia was seen in RSA59(P), RSMHV2(P) and RSMHV2(PP) infected cultures. Experiments were performed three times.

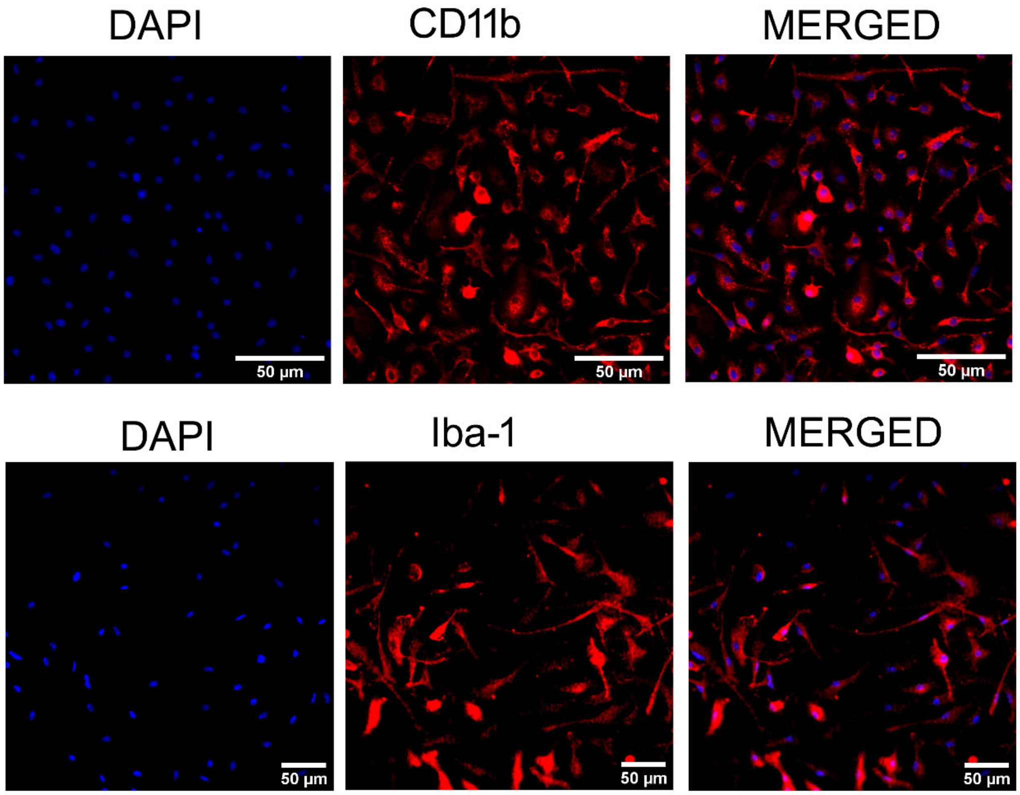

Figure S3. Primary microglia culture immunofluorescent characterized with CD11b and Iba-1 staining. Microglia-enriched cultures were immunolabeled with anti-CD11b (upper panels) and anti-Iba-1 (lower panel) and counterstained with DAPI. Total cells in culture were shown by DAPI (blue) and CD11b and Iba-1 positive cells were shown in red. Corresponding DAPI and microglia markers channels were merged.

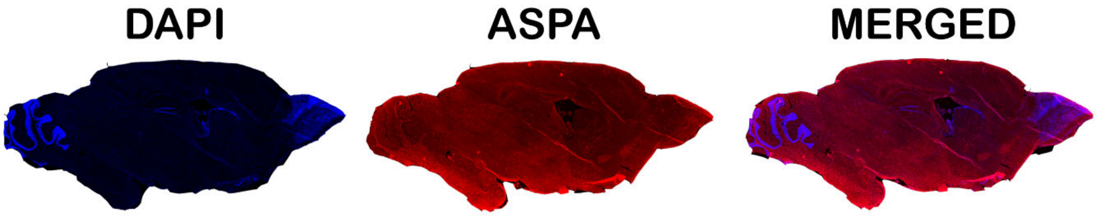

Figure S4. Distribution of oligodendrocytes in whole brain sections**.** 10um thick sections of the brain were immunolabeled with anti-Olig2 (Red) and counterstained DAPI (Blue). The merged panel shows the DAPI channel merged with the Olig2-positive channel.

Table S1. Spreadsheet of the data statistics.

Figure	Viruses	No. of Frames	No. of Cells Per Frame (Average)	No. of Neuron Per Frame (Average)	No. of Infected Cells Per Frame (Average)
Fig.1	RSA59(PP)	12	1110	794	325
	RSA59(P)	5	1077	648	35
	RSMHV2(P)	12	1339	899	88
	RSMHV2(PP)	12	1549	944	99
Fig.2	RSA59(PP)	8	12	5	2
	RSA59(P)	8	13	5	2
	RSMHV2(P)	6	14	5	1
	RSMHV2(PP)	6	13	5	2
Fig.3	RSA59(PP)	9	96	no stain	72
	RSA59(P)	9	121	no stain	52
	RSMHV2(P)	10	102	no stain	54
	RSMHV2(PP)	9	113	no stain	65
Fig.4	RSA59(PP)	9	68	28	7.4
	RSA59(P)	10	63	25	6.4
	RSMHV2(P)	7	56	13	1.2
	RSMHV2(PP)	6	75	12	2.4
Fig.5	RSA59(PP)	13	465	no stain	69
	RSA59(P)	7	410	no stain	33
	RSMHV2(P)	13	432	no stain	73
	RSMHV2(PP)	13	385	no stain	38
Fig.6	RSA59(PP)	7	65	15	0.7
	RSA59(P)	5	68	15	0.25
	RSMHV2(P)	10	61	17	2.4
	RSMHV2(PP)	9	69	11	0.4
Fig.7	RSA59(PP)	20	274	263	16
	RSA59(P)	15	282	276	10
	RSMHV2(P)	20	321	305	67
	RSMHV2(PP)	20	249	244	12
Fig.8	RSA59(PP)	9	223	28	2.5
	RSA59(P)	9	216	25	1.2
	RSMHV2(P)	9	281	29	2.4
	RSMHV2(PP)	9	262	27	2.2
Fig.9	RSA59(PP)	7	75	16	1.8
	RSA59(P)	5	68	14	0.8
	RSMHV2(P)	5	64	15	0
	RSMHV2(PP)	7	57	11	2.1
Fig.10	RSA59(PP)	10	58	57	5
	RSA59(P)	10	62	59	5
	RSMHV2(P)	10	57	56	23
	RSMHV2(PP)	10	56	54	10
Fig.11	RSA59(PP)	11	1831	727	528
	RSA59(P)	11	1754	665	487
	RSMHV2(P)	11	1812	524	41
	RSMHV2(PP)	11	1780	610	387
